# Deregulation of miR-183 and *KIAA0101* in Aggressive and Malignant Pituitary Tumors

**DOI:** 10.3389/fmed.2015.00054

**Published:** 2015-08-10

**Authors:** Magali Roche, Anne Wierinckx, Séverine Croze, Catherine Rey, Catherine Legras-Lachuer, Anne-Pierre Morel, Alfredo Fusco, Gérald Raverot, Jacqueline Trouillas, Joel Lachuer

**Affiliations:** ^1^Centre de Recherche en Cancérologie de Lyon, INSERM U1052/CNRS UMR 5286 Centre Léon Bérard, Lyon, France; ^2^Université Lyon 1, Université de Lyon, Lyon, France; ^3^ViroScan3D, Trévoux, France; ^4^ProfileXpert, SFR-Est, CNRS UMR-S3453, INSERM US7, Lyon, France; ^5^UMR CNRS 5557 UCBL USC INRA 1193 ENVL, Dynamique Microbienne et Transmission Virale, Lyon, France; ^6^Instituto di Endocrinologia ed Oncologia Sperimentale del CNR c/o Dipartimento di Biologia e Patologia Cellulare e Molecolare, Università degli Studi di Napoli “Federico II”, Naples, Italy; ^7^Instituto Nacional de Câncer (INCA), Rio de Janeiro, Brazil; ^8^UMR 5292, Centre de Neurosciences de Lyon, CNRS, INSERM S1028, Lyon, France; ^9^Fédération d’Endocrinologie, Groupement Hospitalier Est, Hospices Civils de Lyon, Lyon, France; ^10^Centre de Pathologie Est, Groupement Hospitalier Est, Hospice Civils de Lyon, Bron, France

**Keywords:** pituitary tumor, PRL tumor, miRNA, integrative genomics, aggressiveness, malignancy

## Abstract

Changes in microRNAs (miRNAs) expression in many types of cancer suggest that they may be involved in crucial steps during tumor progression. Indeed, miRNAs deregulation has been described in pituitary tumorigenesis, but few studies have described their role in pituitary tumor progression toward aggressiveness and malignancy. To assess the role of miRNAs within the hierarchical cascade of events in prolactin (PRL) tumors during progression, we used an integrative genomic approach to associate clinical–pathological features, global miRNA expression, and transcriptomic profiles of the same human tumors. We describe the specific down-regulation of one principal miRNA, miR-183, in the 8 aggressive (A, grade 2b) compared to the 18 non-aggressive (NA, grades 1a, 2a) PRL tumors. We demonstrate that it acts as an anti-proliferative gene by directly targeting *KIAA0101*, which is involved in cell cycle activation and inhibition of p53–p21-mediated cell cycle arrest. Moreover, we show that miR-183 and *KIAA0101* expression significantly correlate with the main markers of pituitary tumors aggressiveness, Ki-67 and p53. These results confirm the activation of proliferation in aggressive and malignant PRL tumors compared to non-aggressive ones. Importantly, these data also demonstrate the ability of such an integrative genomic strategy, applied in the same human tumors, to identify the molecular mechanisms responsible for tumoral progression even from a small cohort of patients.

## Introduction

Endocrine pituitary tumors are some of the most frequent intracranial tumors, and are generally considered benign. Nevertheless, many of these tumors (30–45%) are invasive (1) and some are clinically aggressive with recurrences and resistance to medical treatment (2, 3). In 2004, the World Health Organization (WHO) classification identified the “atypical adenoma” with “uncertain malignancy” (4). Only tumors with metastasis were considered malignant. Our group previously studied tumor progression in prolactin (PRL) tumors, which have been characterized clinically, histologically, molecularly, and genetically (5–7). Recently, a new classification of the five immunohistochemical (IHC) types of pituitary tumors (GH, PRL, ACTH, FSH-LH, and TSH) has been proposed, which introduces a grading that takes the invasion and the proliferation into account (7). However, the molecular events responsible for tumor progression from an aggressive to malignant phenotype remain unknown.

Transcriptomic studies comparing all types of pituitary tumors to normal pituitary have highlighted different deregulated genes and pathways [reviewed in Ref. (8)]. However, as all tumor types were combined, the comparison of gene expression was able to decipher mechanisms involved in tumorigenesis, but not those specifically involved in progression. A recent study coupling transcriptomic and DNA copy number analyses performed on one subtype of pituitary tumor, PRL tumors, has revealed the deregulation of genes connected in a specific signaling pathway that controls cell proliferation, as well as an allelic loss on chromosome 11 specifically associated with aggressiveness and malignancy (5, 6, 8, 9). Nevertheless, the driving role of these alterations during progression toward aggressiveness is still unknown.

To decipher the driving event(s) leading to aggressive and malignant phenotypes of PRL tumors, we explored the global alterations of microRNAs (miRNAs) expression. miRNAs are a class of small non-coding RNAs, between 21 and 25 nt in length, involved in post-transcriptional regulation of gene expression (10). They act by binding to 3′-untranslated regions (3′-UTR) of target mRNAs, causing inhibition of translation or mRNA degradation (10). Several studies have shown that miRNAs are deregulated in many types of cancer and act as tumor suppressors or oncogenes depending on the function of the targeted genes [for review, see Ref. (11, 12)]. Moreover, miRNAs seem to be involved in the regulation of many steps of tumor progression (12, 13) that are defined as hallmarks of cancer (chronic proliferation, resistance to cell death, unlimited replicative potential, induction of angiogenesis, invasion, and metastasis) (14).

Many studies have explored the role of miRNAs in pituitary tumorigenesis by comparing tumoral to normal pituitary [Table S1 in Supplementary Material; (15–29)]. To our knowledge, however, only one study has explored the alteration of miRNAs malignancy (21).

In this study, we have performed a genome-wide analysis of miRNA expression in the same non-aggressive and aggressive PRL tumor samples as those used in earlier analyses (5, 9). Further, we propose a strategy of data filtering to find candidate miRNAs involved in tumor progression toward aggressiveness and malignancy.

## Materials and Methods

### Patients and tumors characteristics

The 26 patients, 13 males and 13 females presenting with hyperprolactinemia without acromegaly, underwent surgery for pituitary tumors. None were operated under dopamine agonist treatment at the time of operation. Twenty-five patients were part of the Hypopronos cohort, previously published in Ref. (6, 7, 9). By histology, all the tumors were sparsely granulated and expressed PRL in almost all cells. Taking into account the invasion of the cavernous or the sphenoid sinus by MRI and the three markers of the cell cycle (mitoses, Ki-67, and p53), the tumors were classified into five grades (1a: non-invasive; 1b: non-invasive and proliferative; 2a: invasive; 2b: invasive and proliferative; and 3: malignant) (7) and split into two groups, non-aggressive (grades 1a; 2a; *n* = 18) or aggressive (grade 2b;*n* = 8), according to the pathological classification and clinical behavior during follow-up. Three tumors (#3, #4, and #5) were considered malignant and classified as grade 3 based on the occurrence of metastasis during follow-up. The main clinical and pathological data are shown in Table 1. For all tumors, fragments were immediately frozen in liquid nitrogen after verification by the pathologist, and kept at −80° until molecular analysis. The same tumors have been used for different molecular analyses (microarrays, PCR, and CGH) recently published by our group (5–7, 9, 30).

**Table 1:** **Clinical and pathological data in 26 patients operated for PRL tumors**.

Tumor number	Sex	Age	Preoperative PRL plasma level	Tumor size	Invasion	Ki-67%	P53%	Tumor grades^a^	Groups^b^	Postoperative events
1*	M	66	2610	Macro	Yes	8	3	2b	A	Recurrence, death
2*	F	54	440	Macro	Yes	4	1.3	2b	A	Recurrence
3*	M	68	4170	Macro	Yes	10	2.3	2b**	A	Recurrence, metastasis, death
4*	M	54	400	Macro	Yes	20	1	2b**	A	Recurrence, metastasis, death
5*	F	31	3000	Macro	Yes	30	10	2b**	A	Recurrence, metastasis, death
6*	F	43	150	Macro	Yes	2.7	0.5	2b	A	Recurrence
25	M	60	690	Giant	Yes	3.6	2.6	2b	A	Recurrence
26	M	53	320	Macro	Yes	5.5	2	2b	A	Recurrence
7*	M	40	1700	Macro	Yes	0	0	2a	NA	Persistence
8*	M	42	1540	Micro	Yes	1.8	0	2a	NA	Persistence
9*	M	42	700	Macro	No	0	0	1a	NA	Remission
10*	F	44	100	Micro	No	0	0	1a	NA	Remission
11*	M	39	4080	Giant	No	1.3	0	1a	NA	Remission
12*	F	30	97	Micro	No	0	0	1a	NA	Remission
13	F	21	900	Macro	No	0	0	1a	NA	Remission
14	F	28	80	Micro	No	0	0	1a	NA	Remission
15	F	36	120	Macro	No	0	0	1a	NA	ND
**16**	F	25	120	Micro	No	0	0	1a	NA	Remission
**17**	F	55	860	Macro	No	0	0	1a	NA	Remission
**18**	F	26	300	Micro	No	0	0	1a	NA	Remission
19	M	26	180	Macro	Yes	0.5	1	2a	NA	Recurrence
20	F	38	105	Micro	Yes	0	0	2a	NA	Recurrence
21	M	27	1000	Giant	Yes	0	0	2a	NA	Persistence
22	M	52	810	Macro	Yes	0	0	2a	NA	Persistence
23	F	73	ND	Macro	Yes	0	0	2a	NA	ND
24	M	45	1000	Giant	Yes	0	0	2a	NA	Persistence

*Tumors used for CGH and transcriptomic profiling in Wierinckx et al. (9); tumors in bold used for miRnome analysis.

**These tumors were grade 2b at the first surgery, and became grade 3 because metastasis appeared during follow-up

*^a^Classification of the tumors according to Trouillas et al. (7)*.

*^b^Tumors split in two groups: aggressive (A) and non-aggressive (NA) according to Wierinckx et al. (9)*.

The study was approved by the ethics committee of Lyon, and informed consent was obtained for each patient according to French law. This authorization was given in the context of a larger French clinical study named HYPOPRONOS (Programme Hospitalier de Recherche Clinique National 27-43).

### Isolation of total RNA

Total RNA from pituitary tumors and normal pituitary was extracted using the miRNeasy Mini kit with DNase treatment (Qiagen, Venlo, Netherlands) according to the manufacturer’s protocol. Total RNA from cell lines was extracted using miRNeasy Micro kit with DNase treatment (Qiagen) according to the manufacturer’s protocol. Total RNA yield was measured by OD260 and the purity confirmed by reaching an A260/A280 ratio of 1.9:2.1 on a Nanodrop ND-1000. The quality was evaluated on nanochips with an Agilent 2100 Bioanalyzer (Agilent Technologies, Palo Alto, CA, USA) according to the manufacturer’s protocol.

### miRNA microarray processing and analysis

Global miRNA expression was assessed in 12 of 26 PRL tumors (8 non-aggressive and 4 aggressive) (Table 1, tumors in bold) using Human miRNA Microarray v3, 8 × 15k from Agilent Technologies. Microarrays were processed as described in the miRNA Microarray System with miRNA Complete Labeling and Hyb Kit version 2.4 September 2011 (available at http://www.genomics.agilent.com). Microarrays were scanned on an Agilent Scanner Type C. Data were extracted using Agilent feature extraction software 10.7. Data files (.txt) from Feature Extraction and then were normalized using spikes in Genespring Software 7.0 (Agilent Technologies). Statistical analysis was performed using Genespring software 7.0 to isolate differentially expressed miRNAs in aggressive–invasive vs. non-aggressive tumors. A miRNA transcript was considered differentially expressed if the difference gave a *p*-value ≤0.05 in the Welch ANOVA parametric test, and showed a minimal 1.5 fold variation. Data are available on the GEO database under the accession number GSE46294. Some of these tumors were previously analyzed for transcriptomic profiling in Ref. (9).

### TaqMan low density array analysis

TaqMan Low Density Array (Applied Biosystems, Carlsbad, CA, USA) allowed testing of a large subset of miRNAs by RT-qPCR in a single assay. Briefly, 500 ng of total RNA was used for cDNA synthesis with the TaqMan microRNA Reverse Transcription kit according to the manufacturer’s protocol. Then, miRNA expression analysis was performed by Real-Time PCR using the TaqMan MicroRNA Array (Applied Biosystems) according to the manufacturer’s protocol and run on a 7900HT Fast Real-Time PCR System (Applied Biosystems). Threshold cycle (Ct) values provided an index of miRNA level. The level of miRNA RNU6 was used as an internal standard. Values were obtained using RQ Manager Software for analysis (Applied Biosystems) and the detection limit was 32Ct. Comparison between samples was made using Genespring software 7.0 (Agilent Technology).

### miRNA RT-qPCR

Single miRNA expression was assessed in cell lines and 26 PRL tumors (18 non-aggressive and 8 aggressive tumors) using TaqMan microRNA assays (Applied Biosystems). Assays with the following identification numbers were used in this study: miR-183 no. 002269; miR-let-7g no. 002282; and RNU6B no. 001093. Briefly, RT primers were multiplexed to obtain mixed RT primers at 62.5 nM each. Then, 40 ng of total RNA was reverse-transcribed using the TaqMan microRNA reverse transcription kit (Applied Biosystems) with the following modifications: primers at a 12.5 nM final concentration, dNTP at 2 nM final concentration, 100 U of MultiScribe Reverse Transcriptase per reaction, RT Buffer 1× final concentration, and 5 U of RNase inhibitor per reaction. The RT program used was 30 min at 16°C, 30 min at 42°C, and 5 min at 85°C. mRNA levels were analyzed by qPCR using TaqMan small RNA assays at 1× final concentration and TaqMan Universal PCR Master Mix II No UNG at 0.5× final concentration, run on a 7900HT Fast Real-Time PCR System with the following cycle: standard mode, 10 min at 95°C then 40 cycles of denaturation at 95°C for 15 s and hybridization and elongation at 60°C for 60 s. Relative miRNA expression was measured using hsa-let-7g (shown to be constant among tumors by microarray) and *RNU6B* as a reference with RQ Manager Software (Applied Biosystems).

### Gene expression analysis

Five hundred nanograms of total RNA were reverse-transcribed using the iScript cDNA synthesis kit (Bio-Rad, Hercules, CA, USA) following the manufacturer’s protocol. The cDNA synthesized was measured by RT-qPCR using FAST SYBR Green Master Mix (Applied Biosystems) following the manufacturer’s recommendations. The PCR program was as follows: 95°C for 20 s then 40 cycles of denaturation at 95°C for 1 s and hybridization and elongation at 60°C for 20 s on a 7900HT Fast Real-Time PCR System. Relative expression analysis was performed using RQ Manager Software (Applied Biosystems) with the *RPL4* gene as a reference. Primers were designed using the NCBI Primer-Blast algorithm. All primers are listed in Table S2 in Supplementary Material.

### Cell culture and transfection

HeLa cells (high proliferating rate) were cultured in DMEM with 10% FBS and 1% penicillin/streptomycin (PS). ZR-75-1 cells (mammary epithelial cancer cell line, low proliferating rate) were cultured in RPMI with 10% FBS, 1% Pyruvate, and 1% PS. Transfection of pre-miR miRNA precursor (Ambion for Life Technologies) was performed using 100 nM final concentration of pre-miRNA precursor with SiPORT NeoFX Transfection Agent (Ambion) following the manufacturer’s instructions. Three pre-miRNA precursors were used: hsa-pre-miR-183 (PM10426), the positive control hsa-pre-miR-1 (AM17150), and the negative control pre-miR miRNA precursor negative control #1 (AM17110). Transfections of plasmids were performed using FuGENE^®^ 6 Transfection Reagent (Promega) following the manufacturer’s instructions.

### Plasmids and constructs

The human pre-mRNA expression construct lenti-miR-183 (MI0000273) and scramble control hairpin in pCDH-CMV-MCS-EF1-copGFP (CD511B-1) were purchased from System Biosciences (SBI, Mountain View, CA, USA). A pcDNA3 plasmid containing ETS2-flag purchased from Addgene (31) was used to express ETS2, with an empty pcDNA3 as a control. Following the supplier’s instructions, the pmirGLO Dual-Luciferase miRNA Target Expression Vector (Promega, Madison, WI, USA) was used to measure the activity of miR-183 matching the target sequence in KIAA0101; sequences were cloned into the 3′-UTR of luciferase (sense, 5′-AAACTAGCGGCCGCTTTGATTATTGGAATGGTGCCATATTGT-3′; antisense, 3′-TTTGATCGCCGGCGAAACTAATAACCTTACCACGGTATAACAGATC-5′), and with mismatch sequence within the seed (sense, 5′-AAACTAGCGGCCGCTTTGATTATTGGAATGGT*AAA*TATTGT-3′; antisense, 3′-TTTGATCGCCGGCGAAACTAATAACCTTACCA*TTT*TATAACAGATC-5′).

### Protein expression analysis

Cells were lysed in RIPA buffer supplemented with a complete protease inhibitor cocktail (Roche). Protein expression was examined by western blot using mouse monoclonal anti-KIAA0101 ab 56773 (Abcam, Cambridge, UK), rabbit polyclonal anti-ETS2 sc-351 (Santa Cruz Biotechnology, Santa Cruz, CA, USA), and monoclonal anti-β-actin clone AC-15 (Sigma Aldrich, St Louis, MO, USA) antibodies for primary detection. Horseradish peroxidase-conjugated rabbit anti-mouse and goat anti-rabbit polyclonal secondary antibodies (Dako, Denmark) were used. Western blots were developed using Luminol (Santa Cruz Biotechnology).

### Proliferation and cell cycle assay

Growth curves were performed by daily counting of viable pre-miR-183 transfected and scramble transfected cells expressing GFP protein using an LSRII FACS flow cytometer (BD Biosciences Europe, Erembodegem, Belgium). For cell cycle distributions, pre-miR-183 transfected and scramble transfected cells were collected and incubated 1 h at 4°C with propidium iodide (0.05 mg/ml) solution containing Non-idet-P40 (0.05%) for proliferation and cell cycle assays. Cells were analyzed using a FACSCalibur flow cytometer (BD Biosciences Europe, Erembodegem, Belgium) and cell cycle distribution was determined using Modfit LT 2.0™ software (Veritysoftware Inc., Topsham, ME, USA). BrdU incorporation assays were performed using the APC-BrdU flow kit (BD Pharmingen), following the manufacturer’s protocol.

### Chromatin immunoprecipitation assays

HeLa cells were fixed in medium with 1% formaldehyde for 20 min and then harvested in SDS lysis buffer (50 mM Tris HCl pH 8.1, 10 mM EDTA, and 1% SDS) with added protease inhibitors. Chromatin was then sonicated using 25 cycles of 30 s ON/OFF on a Diagenode UCD-300 sonicator. Sheared chromatin was pre-cleared using sepharose beads (Sigma Aldrich) followed by overnight incubation with 5 μg anti-ETS2 (sc-351, Santa Cruz Biotechnology) or 5 μg of rabbit anti-mouse IgG (Dako) as a background control. Samples were then incubated with blocked GammaBind G Sepharose Beads (GE Healthcare Life Sciences, Freiburg, Germany) and eluted in elution buffer (0.1M NaHCO_3_, 1% SDS). Untreated chromatin was used as input. Purified DNA was used in qPCR reactions for analysis. All primers are listed in Table S4 in Supplementary Material.

## Results

### miRNAs are globally deregulated in aggressive vs. non-aggressive PRL tumors

In 12 PRL tumors classified according to the new classification (7) and split into two groups (9): aggressive tumors (*n* = 4) and non-aggressive tumors (*n* = 8) (Table 1, tumors in bold), miRNA expression levels were assessed using Human miRNA Microarray V3 (Agilent Technologies) and analyzed with GeneSpring Software 7.0. Signature comparison revealed a down-regulation of 11 miRNAs in aggressive compared to non-aggressive tumors (Table 2). A technical validation of microarrays was performed using TaqMan Low Density Assay (TLDA, Applied Biosystems) on the same samples and 9 of the 11 miRNAs interrogated by the TLDA assays presented the same changes in expression (Table 2).

**Table 2:** **Deregulated miRNA in aggressive vs. non-aggressive tumors**.

Name	Accession^a^	Chromosome	Position	Strand	Cytoband	Agilent microarrays		TLDA assays	
						FC^b^	*p*-Value^c^	FC^b^	*p*-Value^c^
hsa-miR-183	MI0000273	7	129414745-129414854	[−]	q32.2	−2.74	0.018	−2.23	0.0134
hsa-miR-148a	MI0000253	7	25989539-25989606	[−]	p15.2	−2.56	0.030	−2.46	0.0444
hsa-miR-375	MI0000783	2	219866367-219866430	[−]	q35	−2.39	0.013	−2.01	0.0233
hsa-miR-342-3p	MI0000805	14	100575992-100576090	[+]	q32.2	−2.07	0.050	−2.74	0.0424
hsa-miR-23b	MI0000439	9	97847490-97847586	[+]	q22.32	−1.95	0.032	−1.83	0.0091
hsa-miR-744	MI0005559	17	11985216-11985313	[+]	p12	−1.93	0.015	−2.93	0.0009
hsa-miR-98	MI0000100	X	53583184-53583302	[−]	p11.22	−1.92	0.028	−1.72	0.048
hsa-miR-340*	MI0000802	5	179442303-179442397	[−]	q35.3	−1.86	0.015	No probe	
hsa-miR-574-3p	MI0003581	4	38869653-38869748	[+]	p14	−1.84	0.047	−2.12	0.0337
hsa-miR-331-3p	MI0000812	12	95702196-95702289	[+]	q22	−1.83	0.012	−1.87	0.0143
hsa-miR-1280	MI0006437	3	128081008-128081101	[+]	q21.3	−1.57	0.035	No probe	

*^a^Accession number from MirBase*.

*^b^FC, fold change of miRNA expression in A tumors compared to NA tumors*.

*^c^*p*-Value from Student’s test*.

### miR-183 appeared as a main miRNA involved in progression toward aggressiveness and malignancy

**Figure 1:**
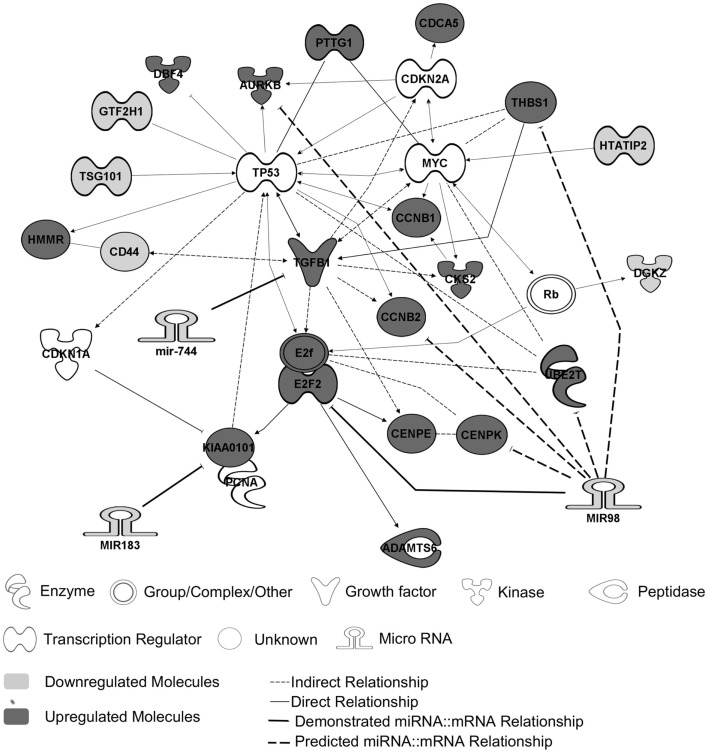
**The “aggressive pathway.”** Up-regulated genes (red) and downregulated genes (blue) are interconnected in a pathway involved in cell cycle control. MiRNAs and their predicted targets are linked by bold lines. The demonstrated targets are linked by solid lines, whereas predicted ones are linked by dotted lines. The genes *HTATIP2*, *DGKZ*, *CD44*, *TSG101*, and *GTF2H1* are downregulated because of an allelic loss (chr11p region) (9).

Previous transcriptomic analysis using Codelink microarrays had been performed on 12 out of the 26 PRL tumors (Table 1, tumors with *) and had revealed the deregulation of specific genes involved in the control of cell cycle and proliferation and interconnected in a pathway specifically up-regulated in aggressive tumors (6, 9). This increased proliferation specifically in aggressive tumors is consistent with the clinical data, the Ki-67 and p53 labeling and the classification recently proposed (7). For the purpose of simplification, herein, we will refer to this pathway as the “aggressive pathway” (Figure 1).

In order to identify miRNA potentially involved in tumoral progression toward aggressive and malignant phenotype, we used the following criteria: (1) miRNA targets (predicted by databases and already published targets) should be linked to the “aggressive pathway”; (2) targets mRNA expression should be inversely correlated to miRNA expression at mRNA level; (3) significant correlation should be observed between miRNA expression and the main molecular markers of cell cycle taking into account in the tumor grading (i.e., Ki-67 and p53).

**Figure 2:**
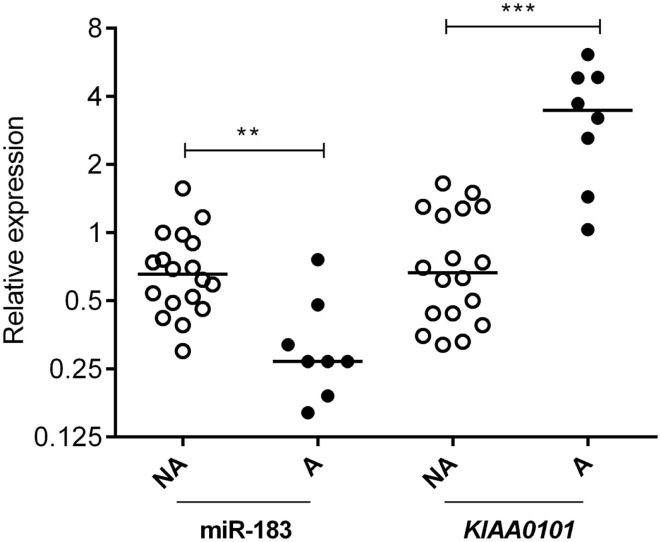
**Expression of miR-183 and its predicted target KIAA0101 in human PRL tumors**. Relative expression of miR-183 and *KIAA0101* target gene was assessed by RT-qPCR in aggressive (A; *n* = 8) and non-aggressive (NA; *n* = 18) human PRL tumors. Statistical relevance was tested using unpaired *t*-test. *p < 0.05; **p < 0.005; ***p < 0.0001.

Two databases (TargetScan and microRNA.org) were used in order to identify known and predicted targets of the 11 deregulated miRNA and lead to the identification of 541 mRNA targets. Among these, only 22 genes presented a variation of expression in aggressive vs. non-aggressive tumors that was inversely correlated to their associated miRNA. Using Ingenuity Pathway Analysis Software on these 22 genes, we found that nine of these genes were linked to the “aggressive pathway” and were predicted to be regulated by four miRNAs (miR-183, miR-340*, miR98, and miR-744) (Table 3). Finally, we assessed the correlation between the four miRNA expression and Ki-67 and p53 labeling in the 26 PRL tumors and it appeared that only miR-183 showed a significant inverse correlation with both cell cycle markers (miR-183:Ki-67 *r* = −0.58, *p* = 0.002; miR-183:p53 *r* = −0.59; *p* = 0.0014) (Table 3). Thus, we focused our attention on miR-183 that is down-regulated in aggressive vs. non-aggressive tumors and its predicted target *KIAA0101* that is significantly up-regulated in aggressive vs. non-aggressive PRL tumors (Figure 2). Finally, KIAA0101 expression was also significantly correlated with Ki-67 (*r* = 0.58; *p* = 0.0018) and p53 (*r* = 0.53; *p* = 0.0054).

**Table 3:** **Predicted target of deregulated miRNA with inverse correlation in expression level**.

miRNA name	Accession^a^	FC^b^	*p*-Value^c^	Gene name	RefSeq	FC^d^	*p*-Value^c^	Correlation^e^ miRNA:mRNA (r; p)	Correlation^f^ miRNA:Ki-67% (r; p)	Correlation^f^ miRNA:P53% (r; p)
miR-183	MI0000273	−2.74	0.018	KIAA0101	NM_014736.4	3.071	0.0002	−0.87; 0.0001	−0.58; 0.002	−0.59; 0.0014
miR-340*	MI0000802	−1.86	0.015	NEK2	NM_002497.2	16.340	0.0077	−0.53; 0.0475	−0.24; ns	−0.19; ns
miR-98	MI0000100	−1.92	0.028	AURKB	NM_004217.2	5.022	0.0002	−0.75; 0.0042	−0.38; 0.0263	−0.19; ns
				CCNB2	NM_004701.2	2.278	0.0084	−0.76; 0.0032	
				CENPK	NM_022145.3	2.468	0.0009	−0.89; 0.0001	
				UBE2T	NM_014176.3	3.810	0.0236	−0.53; 0.0478	
				E2F2	NM_177733.6	3.043	0.0076	−0.56; 0.0340	
miR-744	MI0005559	−1.93	0.015	TGFB1	NM_000660.4	2.640	0.0079	−0.55; 0.0408	−0.31; ns	−0.31; ns

In miRNA name * stands for “minor miRNA” meaning the miRNA originating for the preferentially degraded strand.

*^a^Accession number from Mirbase*.

*^b^FC, fold change of miRNA expression in A tumors compared to NA tumors (26 tumors)*.

*^c^*p*-Value from Student’s test comparing A vs. NA tumors*.

*^d^Fold change of gene expression in A vs. NA tumors (26 tumors)*.

*^e^Correlation coefficient calculated between miRNA expression and their target gene (miRNA:mRNA) using Pearson test. Coefficient (*r*) and *p*-value (*p*) are given in that for each couple*.

*^f^Correlation coefficient calculated between miRNA expression and Ki-67% (miRNA:Ki67%) or P53% (miRNA:P53%) using Spearman test. Coefficient (*r*) and *p*-value (*p*) are given in that for each couple*.

### miR-183 regulates *KIAA0101* by direct interaction with 3′-UTR

**Figure 3:**
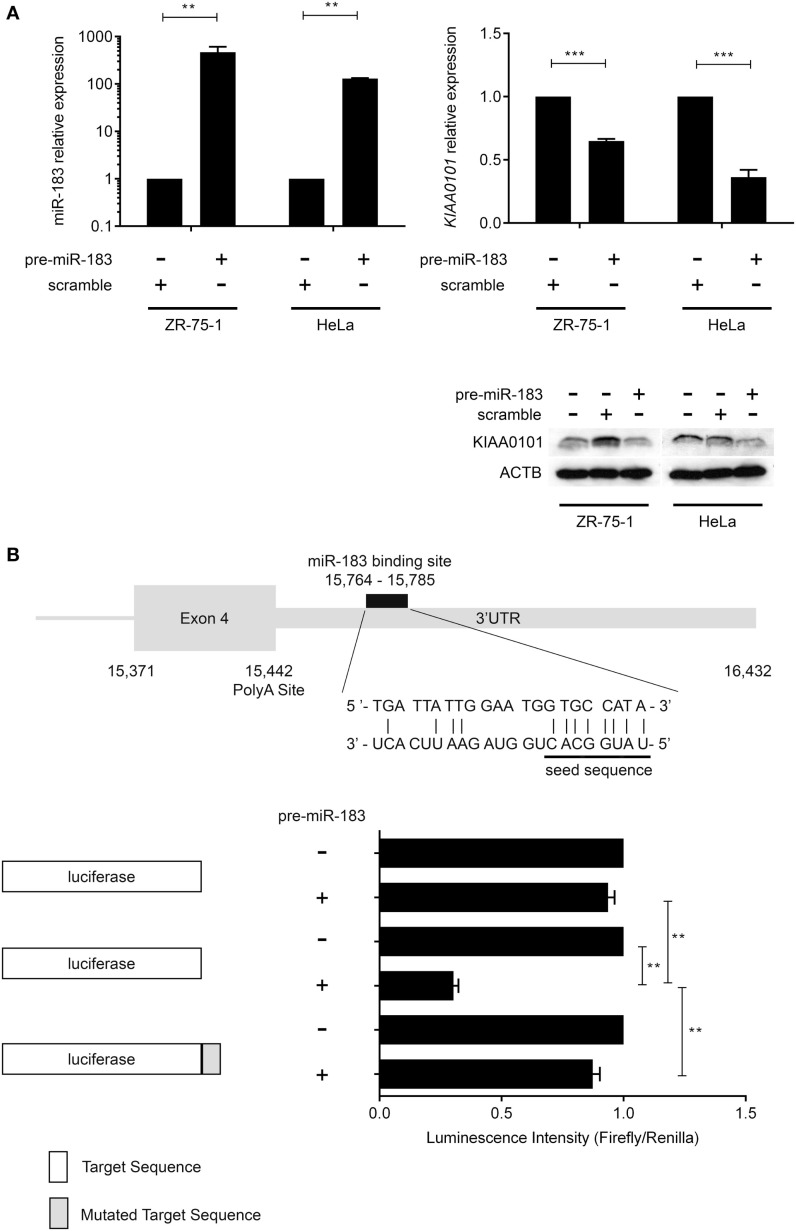
**miR-183 directly regulates *KIAA0101* expression by binding to the 3′-UTR**. **(A)** miR-183 and *KIAA0101* expression levels were assessed by RT-QPCR. Transfection of pre-miR-183 precursor led to a strong increase in miR-183 expression in HeLa and ZR-75-1 cell lines compared to negative control miRNA precursor transfection. Consequently, *KIAA0101* expression was strongly decreased in both cell lines transfected with pre-miR-183 precursor compared to negative control. KIAA0101 protein level decreased in both cell lines overexpressing miR-183 compared to negative control (scramble) transfected cells. **(B)** Predicted binding site of miR-183 in *KIAA0101* 3′ UTR was cloned 3′ of the luciferase sequence (match sequence). As a negative control, a mismatch sequence is the seed was cloned 3′ of the luciferase gene. Moreover normal luciferase was also used. The luciferase assay clearly showed that luciferase activity is decreased only when miR-183 is overexpressed in the presence of match sequence. Statistical relevance was assessed using *t*-test *p < 0.05; **p < 0.005; ***p < 0.0001.

In order to verify miR-183 truly regulate its predicted target, we over-expressed this miRNAs using pre-miR miRNA precursors in HeLa and ZR-75-1 cell lines and measured the effect on the expression levels of the predicted target by RT-qPCR. These cell lines were used because the miRNAs of interest are expressed at very low levels endogenously and present two different proliferation rates (very high with HeLa cells and low with ZR-75-1). Post-transcriptional regulation of *KIAA0101* expression by miR-183 was assessed *in vitro* by monitoring *KIAA0101* mRNA and protein levels in HeLa and ZR-75-1 cell lines 48 h after transfection of pre-miRNA-183. First, we confirmed that transfection of pre-miRNA-183 leads to a strong and significant overexpression of mature miR-183 using TaqMan microRNA Assay (Figure 3A). Following miR-183 overexpression, we observed a strong decrease in *KIAA0101* mRNA and protein levels in both cell lines (Figure 3A). The miR-183 was found to inhibit *KIAA0101* expression by directly interacting with mRNA 3′-UTR as shown by luciferase-reporter assay. Moreover, the specificity of miR-183 binding was assessed using a mutation in the sequence of *KIAA0101* that is bound by miR-183 seed (Figure 3B).

### miR-183 overexpression decreases cell proliferation

**Figure 4:**
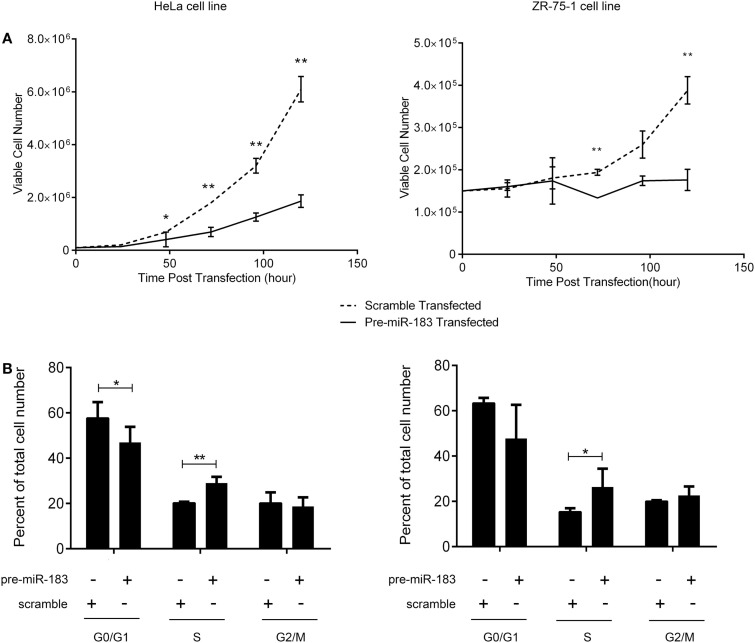
**miR-183 overexpression induces cell accumulation in S phase thus decreasing cell proliferation**. **(A)** HeLa and ZR-75-1 cell lines overexpressing miR-183 showed a significant decrease in cell proliferation compared to scramble control transfected cells. Growth curves were performed by counting viable cells after miR-183 or scramble control transfection every day for 5 days. **(B)** HeLa and ZR-75-1 cell lines overexpressing miR-183 seemed to get trapped in S-phase of the cycle when compared to scramble control transfected cells. Cell cycle analysis was performed by propidium iodide staining of cells 120h after miR-183 or scramble control transfection. Stained cells were analyzed on the LSRII FACS System to assess cell cycle phases. Experiments were performed in triplicate. Statistical relevance was assessed using *t*-test *p < 0.05; **p < 0.005; ***p < 0.0001.

The criteria used to identify candidate miRNAs involved in aggressiveness were strongly linked to proliferation as it is the main difference between non-aggressive and aggressive PRL tumors. Thus, we assessed the effect of miR-183 overexpression on HeLa and ZR-75-1 cell lines. We performed growth curves on three independent experiments overexpressing miR-183 in each cell line. As shown in Figure 4A, miR-183 overexpression in HeLa and ZR-75-1 cell lines led to a significant slowdown in cell growth. Moreover, evaluation of the distribution of cell cycle phases at 120 h after miR-183 overexpression, using FACS analysis with propidium iodide labeling, showed that S phase tended to be delayed, which could explain the decrease of proliferation rate (Figure 4B), this was confirmed by a BrdU incorporation assay (data not shown). These results strongly suggest the involvement of miR-183 loss in control of proliferation and thus in the progression toward aggressive phenotype.

### MiR-183 expression is controlled by the transcription factor ETS2

**Figure 5:**
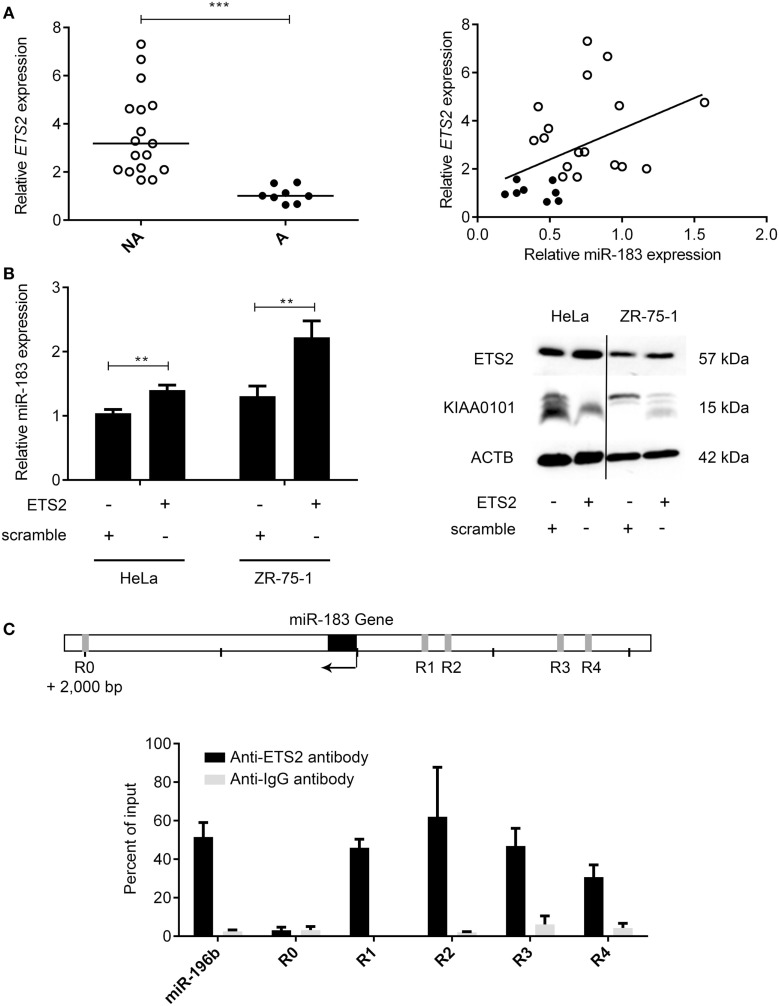
**The transcription factor ETS2 regulates miR-183 expression**. **(A)** *ETS2* relative expression was assessed in 20 non-aggressive and 6 aggressive human PRL tumors by RT-qPCR and a correlation graph was made between miR-183 and *ETS2* expression levels. **(B)** HeLa and ZR-75-1 cell lines overexpressing *ETS2* showed an increased miR-183 expression compared to scramble control transfected cells. Moreover, in ZR-75-1 cells expressing ETS2, KIAA0101 was decreased at the protein level as compared to scramble control transfected cell. **(C)** ChIP assays using antibody directed against *ETS2* showed a significant enrichment of *ETS2* binding sites in the miR-183 regulatory regions (R1–R4) by qPCR compared to background control. Regions containing no *ETS2* binding sites (R0) showed no enrichment. Statistical relevance was assessed using *t*-test *p < 0.05; **p < 0.005; ***p < 0.0001.

The main mechanisms revealed to date underlying the deregulation of miRNA expression in cancers are changes in chromosome copy number or DNA methylation (32). The analysis of these mechanisms in PRL tumors showed no significant differences between aggressive and non-aggressive tumors for miR183 miRNA analyzed (data not shown). We therefore decided to search for a transcription factor that could potentially be involved in regulating the expression of this miRNA. Computational analysis of miR-183 regulatory region using PROMO algorithm (33, 34) showed that 60 transcription factors could potentially regulate miR-183 (Table S4 in Supplementary Material). Correlations between the expression of transcription factors and miRNA were calculated using Spearman test and only ETS2:miR183 (*r* = 0.43, *p* = 0.0167) showed significant correlation in the pituitary tumors studied, moreover, *ETS2* showed a significant down-regulation at the mRNA level in the aggressive tumors compared to the non-aggressive ones (Figure 5A; Table S4 in Supplementary Material). We then tested the effect of overexpressing *ETS2* in HeLa and ZR-75-1 cell lines on miR-183 and its target KIAA0101. As shown in Figure 5B, overexpression of *ETS2* in both cell lines significantly increased miR-183 expression. Moreover, exogenous expression of ETS2 led to a decrease of KIAA0101 at the protein level potentially through the observed increase in expression of miR-183. Finally, chromatin immunoprecipitation experiments revealed that ETS2 directly regulates miR-183 expression by binding its regulatory regions (Figure 5C). All these results seemed to show that ETS2 is involved in the regulation of miR-183 and that its down-regulation in aggressive PRL tumors could explain, at least in part, the decrease of miR-183 expression.

## Discussion

This study explores the role of miRNA in the progression of 26 PRL tumors with different behaviors, including 3 carcinomas and attempts to characterize the hierarchical molecular events leading to malignancy by integrating miRNA signatures with previously obtained transcriptomic and genotyping data from the same tumor samples. To reduce the complexity of the analysis, we chose to focus on genes connected to a signaling pathway that we have previously highlighted as up regulated in aggressive PRL tumors (8, 9). While various studies on miRNAs have compared miRNA deregulation between normal and tumoral pituitary tissues (Data S1 in Supplementary Material) thus highlighting miRNA involvement in pituitary tumorigenesis, to our knowledge, only one other study has investigated miRNA in tumoral progression by comparing the differences between adenomas and carcinomas in ACTH pituitary tumors (21). To assess the role of miRNA deregulation in the progression of PRL tumors, we performed the first global miRNA expression analysis in aggressive and non-aggressive PRL tumors. Eleven miRNAs were identified as significantly deregulated between the two tumor groups. Some of these deregulated miRNA have been described in studies on pituitary tumorigenesis (tumoral vs. normal pituitary tissues) (Table S1 in Supplementary Material). For example, miR-98 and miR-23b are involved in tumorigenesis of pituitary tumors and are down-regulated in GH tumors compared to normal pituitary samples and can directly inhibit *HMGA2* expression (35). Up-regulation of the *HMGA2* gene has a critical role in pituitary tumorigenesis (24, 25, 29, 36–38). Interestingly, while no change in *HMGA2* expression has been observed between aggressive and non-aggressive PRL tumors, comparison with normal pituitary showed a significant increase in *HMGA2* mRNA levels, consistent with a decrease in miR-23b and miR-98 expression (data not shown). Similarly, miR-744 was down-regulated in macro GH-adenomas vs. micro GH-adenomas, suggesting an anti-tumoral role for this miRNA in GH pituitary tumors (20). Among the predicted targets of the deregulated miRNAs, *E2F2* and *TGF*β*1* have already been shown to be regulated by miR-98 and miR-744, respectively (39, 40). E2F2 is involved in the control of the G1/S phase transition and S phase progression and its overexpression has been described in many cancers, in which it is thought to promote cell proliferation (41). Moreover, the E2F family, and especially E2F1, is overexpressed in GH adenomas (24). Interestingly, E2F2 overexpression, in contrast to E2F1, does not activate replicative senescence pathways (42, 43). The *TGF*β*1* pathway was described to be decreased during pituitary tumorigenesis, especially in non-functioning adenoma, suggesting a tumor suppressor role for this gene (22). Nevertheless, a role is emerging for TGF-beta in driving tumoral progression through different mechanisms that favor immunosuppressive activity, pro-angiogenic effects, and epithelial-to-mesenchymal transition, the latter of which is important in the development of metastasis (44). Consequently, it would be interesting to explore the role of *TGFB1* in PRL pituitary tumor aggressiveness and malignancy.

In order to identify candidate miRNAs involved in progression to aggressiveness and malignancy, we used criteria based on the main differences between aggressive and non-aggressive PRL pituitary tumors, which are linked to proliferation. Indeed, pathological classification showed that invasion and cell cycle markers (Ki-67, p53, and mitosis) are able to discriminate tumor grade and to predict tumor behavior (7). Moreover, we have previously described genes over-expressed in aggressive as compared to non-aggressive tumors that have a diagnostic and/or prognostic ability (5, 6). Using these criteria, we highlight that miR-183 is significantly down-regulated in aggressive vs. non-aggressive tumors. Interestingly, miR-183 has been found to be down-regulated in neuroblastoma (45), pancreatic cancer (46), lung cancer (47, 48), ovarian cancer (49), and osteosarcoma (50, 51) when compared to corresponding normal tissues. It has been shown in several studies that overexpression of miR-183 in corresponding cell lines inhibits cell growth (47, 49, 51). These data are consistent with our study corroborating the decrease in cell proliferation of HeLa and ZR-75-1 cells after transfection with miR-183. As the oncogenic or tumor suppressor role of miRNA depends on the messenger RNA they target, we applied an integrative approach combining data on miRNA global expression with those obtained previously by target prediction algorithms and transcriptomic analysis on the same human PRL tumors. Our integrative analysis has identified *KIAA0101* as a new putative target for miR-183. *In vitro*, we show that miR-183 decreases the expression of *KIAA0101* at both the mRNA and protein levels by directly binding to the 3′-UTR. *KIAA0101* is overexpressed in aggressive vs. non-aggressive PRL tumors, and moreover, *KIAA0101* expression is significantly correlated with Ki-67 and p53 labeling. This is consistent with previous studies showing that *KIAA0101* overexpression is often correlated with tumoral grade and lower survival in hepatocellular carcinoma (52), human non-small cell lung cancer (53), adrenocortical tumors (54), gastric cancer (55), and breast cancer (56). Inhibition of *KIAA0101* by shRNA leads to a strong inhibition of cell proliferation and particularly inhibition of S-phase progression (57). Consistent with these findings, we demonstrate that miR-183-mediated *KIAA0101* silencing decreases HeLa and ZR-75-1 proliferation by deregulating cell cycle progression. Finally, it is interesting to note that *KIAA0101* is involved in the inhibition of p53-p21 cell cycle arrest/replicative senescence activating pathways (55, 58, 59). Thus, the increase in *KIAA0101* expression in aggressive and malignant tumors is consistent with the model of pituitary tumor progression proposed by Melmed, which posits that an inhibition of replicative senescence is necessary for tumor progression (60).

To explain the decrease in miR-183 expression in aggressive PRL tumors, we first analyzed genetic alterations and DNA methylation in regulatory region but could not detect any differences between aggressive and non-aggressive tumors at this locus (data not shown). We then analyzed the deregulation of transcriptional activators of miR-183. Among all of the transcription factors with potential involvement in the regulation of miR-183, only *ETS2* expression correlates with miR-183 expression. Indeed, we demonstrate in this study that *ETS2* activates miR-183 expression by directly binding its regulatory regions. The role of *ETS2* in tumoral progression is controversial. Nevertheless, it has been shown that KO-*ETS2* in intestinal stem cells increases colonic tumor frequency (61). Moreover, *ETS2* overexpression activates p53-dependent apoptosis in the context of Down’s syndrome (62). Finally, in the context of pituitary adenoma, ETS2 is involved in inhibition of PRL gene expression (63), and therefore, the decrease of ETS2 mRNA in PRL tumors is consistent with the observed hyperprolactinemia. In agreement with these data, we found *ETS2* to be down-regulated in aggressive vs. non-aggressive human PRL tumors. Together, these data suggest a role for ETS2 down-regulation in the decreased miR-183 expression observed in the same tumors.

**Figure 6:**
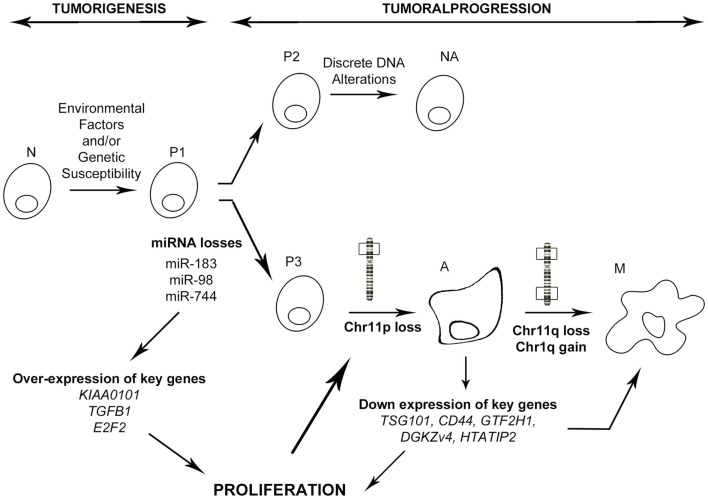
**Model of PRL tumoral progression toward an aggressive phenotype**. N, normal cell; P1–3, predisposed cells 1–3; NA, non-aggressive tumors; A, aggressive tumors; M, malignant tumors (carcinomas). As described in the main text, miRNA down regulation induces a specific progression toward a malignant phenotype involving secondary genetic alterations, whereas absence of miRNA deregulation leads to a non-aggressive phenotype.

In conclusion, integration of the miRNA signature with transcriptomic profiling and clinical–pathological features has allowed us to identify one miRNA and its new target which are strongly involved in PRL tumor progression toward aggressiveness and malignancy. In combination with our previous studies, we propose that the down-regulation of specific miRNAs such as miR-183, perhaps through the inhibition of transcription activators, leads to the overexpression of some key genes connected in the “aggressive pathway” that is specifically up regulated in aggressive tumors. This deregulation of the cell cycle would induce an uncontrolled proliferation that allows the acquisition of advantageous alterations, such as the loss of chromosome 11p, observed specifically in aggressive and malignant PRL tumors (9). This chromosomal alteration would in turn impact the expression of key genes leading to the over-activation of cell proliferation and acquisition of supplementary alterations specific to the malignant phenotype (Figure 6). Finally, we demonstrated that integrative genomic strategy applied in the same human tumors can identify candidate genes and the associated molecular mechanisms responsible for tumor progression, even from a small cohort of patients.

## Author Contributions

JT and GR were responsible of the clinical aspects of the study (sample collection, pathological classification, etc.). JL, MR, JT, GR, CL-L, AW, and AF designed the study, wrote, and revised the manuscript. Final version of the manuscript was approved by all the authors. MR, SC, CR, and APM were involved in data acquisition. APM and MR were involved in analysis and interpretation of data. CL-L, APM, and AF contributed to essential reagents or tools. Finally, all authors agree for all aspects of the work.

## Conflict of Interest Statement

The authors declare that the research was conducted in the absence of any commercial or financial relationships that could be construed as a potential conflict of interest.

## Supplementary Material

The Supplementary Material for this article can be found online at http://journal.frontiersin.org/article/10.3389/fmed.2015.00054
